# EHMTI-0395. Hypnotic relaxation vs amitriptyline for tension-type headache: let the patient choose

**DOI:** 10.1186/1129-2377-15-S1-I3

**Published:** 2014-09-18

**Authors:** Y Ezra, M Gotkine, S Goldman, HM Adahan, T Ben-Hur

**Affiliations:** 1 Neurology, Soroka University Medical Center, Beer Sheva, Israel; 2 Neurology, Hadassah University Medical Center, Jerusalem, Israel; 3 Internal medicine, Hadassah University Medical Center, Jerusalem, Israel; 4 Pain Rehabilitation, Shiba Medical Center, Ramat Gan, Israel; 5 Neurology, Hadassah University Medical Center, Jerusalem, Israel

## Introduction

Although both pharmacological and behavioral interventions may relieve tension-type headache, data are lacking regarding treatment preference, compliance, and feasibility of behavioral intervention in a neurological outpatient clinic setting.

## Aims

To describe patient choice, compliance, and outcome in a neurological clinic where patients are given the choice of the approach they wish to pursue.

## Methods

Patients presenting to the headache clinic with a diagnosis of tension-type headache, were given the choice of amitriptyline (AMT) treatment or hypnotic relaxation (HR), and were treated accordingly. Patients were given the option to cross-over to the other treatment group . HR was performed during standard length neurology clinic appointments by a neurologist . Follow-up interviews were performed between 6 and 12 months following treatment initiation to evaluate compliance, headache frequency or severity, and quality-of-life.

## Results

98 patients were enrolled, 92 agreed to receive prophylactic therapy. 53 (57.6%) patients chose HR of which 36 (67.9%)initiated this treatment, 39 (42.4%) chose AMT of which 25 (64.1%) initiated therapy. 74%of the patients in the HR group and 58% of patients in the AMT group had a 50% reduction in the frequency of headaches (P=.16). At the end of the study , 26 patients who tried HR compared with 10 who tried AMT continued receiving their initial treatment.

## Conclusions

HR was a more popular choice among patients. Patients choosing HR reported greater amelioration than those choosing AMT and were found to have greater treatment compliance. HR practiced by a neurologist is feasible in a standard neurological outpatient clinic.

No conflict of interest.

